# Dynamic Spatiotemporal Expression Pattern of the Senescence-Associated Factor p16Ink4a in Development and Aging

**DOI:** 10.3390/cells11030541

**Published:** 2022-02-04

**Authors:** Hasan Safwan-Zaiter, Nicole Wagner, Jean-François Michiels, Kay-Dietrich Wagner

**Affiliations:** 1 The National Center for Scientific Research (CNRS), The National Institute of Health and Medical Research (INSERM), iBV, Université Côte d’Azur, 06107 Nice, France; Hasan.Safwan-Zaiter@unice.fr; 2 Department of Pathology, CHU Nice, 06107 Nice, France; michiels.jf@chu-nice.fr

**Keywords:** aging, endothelial cells, development, liver, heart, brain, kidney, senescence, SASP

## Abstract

A plethora of factors have been attributed to underly aging, including oxidative stress, telomere shortening and cellular senescence. Several studies have shown a significant role of the cyclin-dependent kinase inhibitor p16ink4a in senescence and aging. However, its expression in development has been less well documented. Therefore, to further clarify a potential role of p16 in development and aging, we conducted a developmental expression study of p16, as well as of p19ARF and p21, and investigated their expression on the RNA level in brain, heart, liver, and kidney of mice at embryonic, postnatal, adult, and old ages. P16 expression was further assessed on the protein level by immunohistochemistry. Expression of p16 was highly dynamic in all organs in embryonic and postnatal stages and increased dramatically in old mice. Expression of p19 and p21 was less variable and increased to a moderate extent at old age. In addition, we observed a predominant expression of p16 mRNA and protein in liver endothelial cells versus non-endothelial cells of old mice, which suggests a functional role specifically in liver endothelium of old subjects. Thus, p16 dynamic spatiotemporal expression might implicate p16 in developmental and physiological processes in addition to its well-known function in the build-up of senescence.

## 1. Introduction

Aging is characterized by the gradual continuous decline of functions of cells, tissues, and the whole organism [1]. This age-related functional degeneration affects each organism that passes through developmental phases up to aging, as it is experienced by single cellular and multicellular organisms [2]. In mammals, aging is associated with a variety of pathologies and has been classified as the leading predictive factor of many chronic diseases that account for the majority of morbidity and mortality worldwide [3]. These diseases include neurodegenerative (Alzheimer’s and Parkinson), cardiovascular, pulmonary, renal, and bone disorders, and cancers [4,5,6,7,8,9]. What makes aging a common risk factor is the fact that it arises from molecular mechanisms and pathological pathways that are cornerstones for the development of all these diseases. This includes oxidative stress and overproduction of reactive oxygen species, overproduction of inflammatory cytokines, activation of oncogenes, DNA damage, telomere shortening, and, consequently, accumulation of senescent cells [10,11,12,13,14,15].

Cellular senescence is a stress response defined as an irreversible arrest of cellular proliferation that results from experiencing potentially oncogenic stress [16]. Senescence was first discovered in primary cell culture in which cells exhibited a replicative senescence after extended period of growth which was termed the Hayflick’s limit [17,18]. Senescent cells are usually characterized by phenotypic changes, morphological and biochemical, and adopt a secretory phenotype known as the senescence-associated secretory phenotype (SASP) [3,19,20,21]. Morphologically, senescent cells are usually larger than normal ones and exhibit a flattened shape, sometimes with multi-nuclei. However biochemically, these cells show a differential expression profile especially for some genes which rendered them as senescence fingerprints. Senescence-associated β-galactosidase, is an enzyme that is upregulated in senescent cells, and which acts as senescence biomarker [22]. Moreover, ectopic expression or upregulation of several genes has been identified, which includes augmented secretion of proinflammatory cytokines, proteases, and growth factors, which are all together termed the SASP [23,24,25]. A variety of causes underly the induction of cellular senescence; this includes oncogenic stress, telomere shortening, mitogenic signals, genomic DNA damage, epigenomic modifications, and tumor suppressor gene dysregulation [26,27,28,29,30,31,32,33].

Two major pathways have been identified to generate and maintain senescence, representing the intrinsic arm of cellular senescence. The key regulatory proteins of these pathways are the cell cycle regulators p16Ink4a (afterwards termed p16), p19Arf (afterwards p19), and p21 in addition to p53 and retinoblastoma protein (pRB). p21 acts mainly as a downstream effector of p53, and p16 is an upstream regulator of pRB via inhibition of cyclin-dependent kinases Cdk4 and Cdk6 [34,35,36,37,38]. Based on their action in regulating the cell cycle, p16, p19, and p21 were associated with cancer, aging, senescence, regeneration, and tumor suppression [21,35,39]. Expression of p19and p21 in embryonic development has been described [21,40,41,42,43], while little is known about the expression of p16 during development [44,45,46]. Therefore, we investigated p16, p19, and p21 RNA expression and p16 protein localization in several organs during embryonic and postnatal development as well as in adult and old mice.

## 2. Materials and Methods

### 2.1. Mice and Tissue Preparation

All animal work was conducted according to national and international guidelines and was approved by the local ethics committee (PEA-NCE/2013/106). 

Timed pregnant mice (NMRI and C57BL/6) were purchased from Janvier Labs (Le Genest-Saint-Isle, France). The day of vaginal plug was considered embryonic day (E) 0.5. Pregnant mice were sacrificed by cervical dislocation at the indicated time points. Embryonic tissues were dissected, and tissues were used to prepare RNA. The day of birth was considered postnatal day (P) 0.

### 2.2. Mouse Tissue Samples, Histology, and Immunohistology

For immunohistochemistry, collections of paraffin-embedded whole embryos were used up to E18.5; for later stages, hearts, livers, kidneys, and brains were dissected. Samples from at least three different animals per time point were analyzed. Three-micrometer paraffin sections were used for histological and immunohistological procedures. For p16 immunohistology, after heat-mediated antigen retrieval and quenching of endogenous peroxidase activity, the antigen was detected after antibody application (1:500 dilution, p16 mouse monoclonal antibody, clone 2D9A12; ab54210, Abcam, Cambridge, UK,; additionally for some samples, a p16 mouse monoclonal antibody, clone 1E12E10, MA5-17142, Thermo Scientific, Courtaboeuf, France) using the M.O.M peroxidase kit from Vector (Vector Laboratories, PK-2200, Burlingame, CA, USA.) following the manufacturer’s instructions. Avidin/Biotin blocking was performed using a kit from Vector (SP-2001). Diaminobenzidine (DAB) served as substrate (Sigma, Saint-Quentin-Fallavier, France). Sections were counterstained with hematoxylin (Dako, Trappes, France) [47,48]. Omission of the first antibody served as a negative control, and additional controls were livers from p16 knockout mice. Slides were photographed using a slide scanner (Leica Microsystems, Nanterre, France) or an epifluorescence microscope (DMLB, Leica, Germany) connected to a digital camera (Spot RT Slider, Diagnostic Instruments, Sterling Heights, MI, USA). For immunofluorescence double-labelling of mouse livers, anti-CD31 rabbit monoclonal antibody (1:2000 dilution, clone EPR17259, Ref: ab225883) from Abcam was combined with the mouse monoclonal anti-p16 antibody (Abcam) using Alexa Fluor 594 donkey anti rabbit and Alexa-Fluor 488 donkey anti mouse secondary antibodies (Jackson ImmunoResearch, Newmarket, Suffolk, UK) [49]. Negative controls were obtained by omission of first antibodies. Images were taken using a confocal ZEISS LSM Exciter microscope (Zeiss, Jena, Germany).

### 2.3. RNA Isolation, Reverse Transcription, and Quantitative PCR

Using the Trizol reagent (Thermo Scientific, Courtaboeuf, France), total RNA was isolated from brain, heart, liver, and kidneys of four different samples each at different stages of development (embryonic day 10.5, 12.5, 14.5, 16.5, and 18.5; postnatal days 1, 7, 21, 3 months, and 16–18 months) [50]. For E10.5 and E12.5, tissues from 7 embryos each were pooled per sample. For E14.5 and E16.5, organs from 4 embryos were used per sample. First-strand cDNA synthesis was performed with 500 ng of total RNA using the Thermo Scientific Maxima First Strand cDNA Synthesis Kit (#K1672, Thermo Scientific, Courtaboeuf, France), which contains DNase I, RNase inhibitor, oligo (DT) and random hexamer primers. The cDNAs were diluted 10 times in nuclease free water. Two microliters of the diluted reaction product were taken for real-time RT-PCR amplification which was performed using a StepOne Plus thermocycler (Thermo Scientific) and the PowerUp SYBR^®^ Green Master Mix (#A25742, Thermo Scientific) or EurobioGreen Mix (GAEMMX02H, Eurobio, Les Ulis, France). For each sample, expression of the housekeeping genes *Gapdh, Rplp0, and β-actin* was determined. Three independent housekeeping genes were used as expression for each gene might vary under different experimental conditions [51,52]. Expression for each sample was calculated by subtracting the mean value of housekeeping gene Ct’s from the gene of interest Ct using the ΔCt method [47,48,50,52,53,54,55,56,57,58]. Afterward, relative gene expression values were obtained by normalization of each sample against the mean value of all samples at E10.5 to determine differences between the organs and time points investigated. The mean value of all samples at E10.5 was set to 1 for easier illustration as described [50]. Primer sequences are listed in Table 1.

### 2.4. Endothelial Cell Magnetic-Activated Cell Sorting (MACS)

Kidneys, livers, hearts, and brains were isolated from four adult (3 months) mice and four old (18 months) mice each. Organs were minced and afterward digested with 0.1 mg/mL of DNase I (10104159001, Roche Diagnostics, Mannheim, Germany) and 1 mg/mL of Collagenase A (11088793001, Roche) in 10 mL of DMEM culture media (ThermoScientific) for 1 h at 37 °C. Digested samples were passed through 70-μm filters (SmartStrainers, 130-098-462, Miltenyi Biotec, Paris, France), centrifuged, and washed twice with PBS containing 2% fetal calf serum (FCS) and 0.5 mM of EDTA (ThermoScientific). Cells were re-suspended in 90 μL of the same buffer (PBS + FCS + EDTA)/10^7^ cells. Endothelial cells were labelled by adding 10 μL/10^7^ cells of magnetic microbead-associated anti-CD31 antibody (130-097-418, Miltenyi) at 4 °C for 15–30 min. Cells were separated via LS column (130-042-401, Miltenyi) pre-washed with 3 mL of PBS + FCS + EDTA and attached to a MidiMACS separator magnet (130-042-302, Miltenyi). Non-endothelial cells were eluted by washes with 3x 3 mL of PBS + FCS + EDTA. Afterward, endothelial cells were eluted by removing the LS columns from the magnetic field and flushing with 6 mL of PBS + FCS + EDTA. Eluted cells were separated as 1/3 for RNA extraction (see above) and 2/3 for protein extraction and quantification.

### 2.5. Protein Isolation, Quantification, and Western Blot

After endothelial cell sorting as described above, 2/3 of each endothelial and organ cells were taken from the total cell suspension. Cells where centrifuged at 3000 rpm for 10 min at 4 °C. Then, cells were incubated with 100 μL and 150 μL of RIPA buffer (Sigma) for endothelial and organ cells, respectively, and kept on ice for 30 min. Afterwards, samples were agitated overnight at 4 °C. The next day, the tubes were centrifuged at 16,000 rpm for 30 min at 4 °C. The total protein containing supernatant was recovered and stored at −80 °C.

Proteins were quantified by colorimetric BCA assay according to manufacturer’s instructions (Uptima, Montluçon, France). Samples were diluted 20 times in distilled water and loaded in triplicates of 10 μL each, in transparent 96-well plates. In addition, BSA standards ranging from 0 to 2 mg were loaded in triplicates (10 μL). Absorbance was measured at a wavelength of 560 nm in a plate spectrophotometer (Biorad, Marnes-la-Coquette, France).

For Western blotting, 60 μg of protein in Laemmli buffer was denatured at 95 °C for 5–10 min. Samples were loaded on acrylamide gels (acrylamide/bisacrylamide 37.5/1) and set for electrophoresis. Afterward, proteins were transferred to PVDF membranes (162-0177, Biorad), and the membranes were blocked with 5% milk for 1 h (232100, Difco Skim Milk). p16 was detected using a rabbit monoclonal anti-p16 (Abcam; ab211542) diluted 1:2000 in PBS + 0.05% Tween 20 + 2.5% milk powder (overnight, 4 °C), followed by anti-rabbit peroxidase-labeled secondary antibody addition (Vector Laboratories) diluted 1:2000 in PBS + 0.05% Tween 20 + 2.5% milk powder for 1 h. Then, the chemiluminescence signal was obtained by incubation with the enzyme-specific substrate (RPN2235, Amersham, ECL Select Western blotting detection reagent). Afterward, the membrane was stripped by application of 10 mL of stripping buffer for 15 min (ST010, Gene Bio-Application L.T.D., Kfar-Hanagid, Israel) and washed 5x 5 min with distilled water before a second identical blocking step with milk for the detection of Gapdh as housekeeping protein. A rabbit monoclonal anti-Gapdh antibody (Abcam; ab181602) was used, and the signal was generated with same secondary antibody and substrate mentioned above.

### 2.6. Statistics

Data are expressed as means ± standard error of the mean (S.E.M.). Statistical differences were assessed by analysis of variance (ANOVA) followed by the Bonferroni post-hoc test (Graph Pad Instat, GraphPad Software, Inc., San Diego, CA, USA). A *p*-value < 0.05 was considered to reflect statistical significance.

## 3. Results

### 3.1. p16Ink4a, p19, and p21 mRNA Expression during Embryonic Development and Postnatal Stages in Different Organs

**Figure 1:**
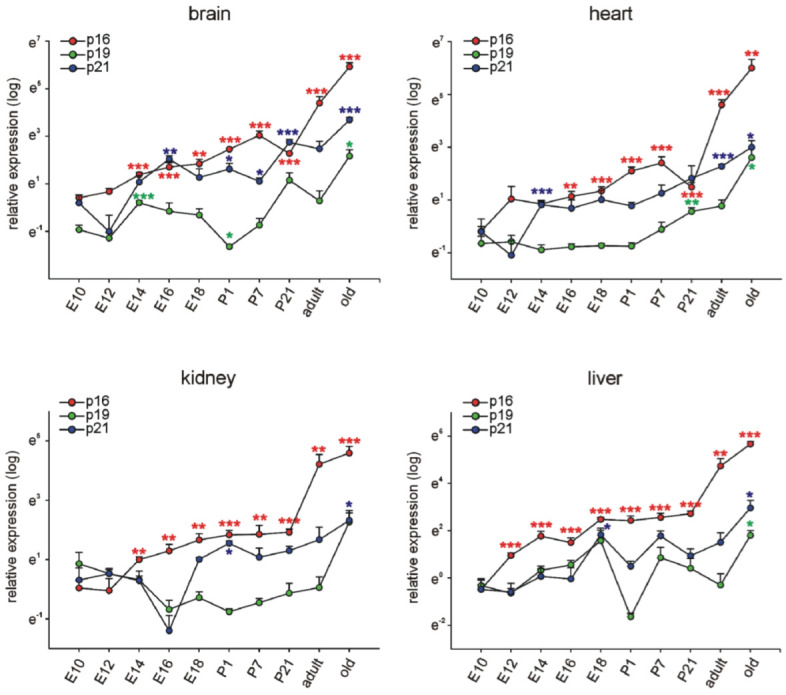
p16, p19, and p21 are differentially expressed during development and adulthood. Quantitative RT-PCRs for p16, p19, and p21 in mouse brains, hearts, kidneys, and livers at different time-points of development and in adulthood (*n* = 4 each, the four samples for E10.5 were each pooled from 7 organs, at E12.5, and 14.5 the four samples were pooled from four organs each). E: embryonic day, P: postnatal day, adult: 3 months of age, old: 16–18 months of age. Expression of each gene was normalized to the respective *Gapdh, actin*, and *Rplp0* expression. Next, the average of all organs and samples at E10.5 was calculated. Individual samples were then normalized against this average value (see Materials and Methods for details). Significance was tested for all time points between E10.5 and 18 months. Data are mean ± SEM. * *p* < 0.05, ** *p* < 0.01, *** *p* < 0.001.

**Figure 2:**
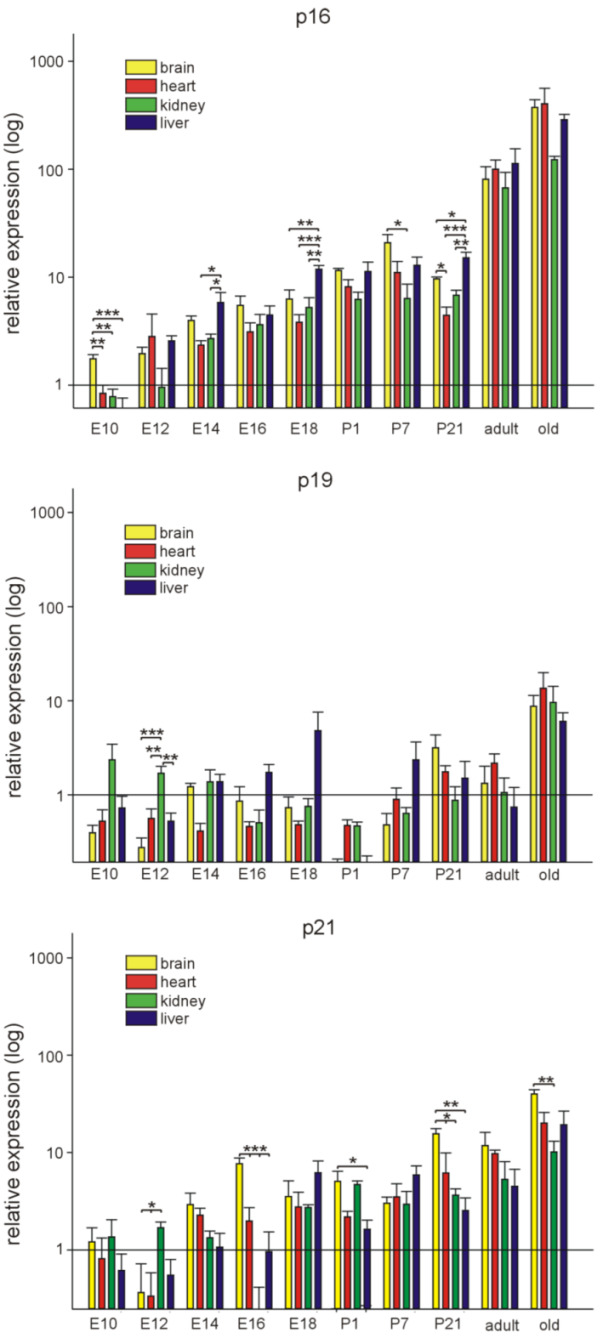
Differential spatiotemporal expression of p16, p19, and p21. Quantitative RT-PCRs for p16, p19, and p21 in mouse brains, hearts, kidneys, and livers at different time points of development and in adulthood (*n* = 4 each, the four samples for E10.5 were each pooled from 7 organs, at E12.5 and 14.5 the four samples were pooled from four organs each). E: embryonic day, P: postnatal day, adult: 3 months of age, old: 16–18 months of age. Expression of each gene was normalized to the respective *Gapdh, actin*, and *Rplp0* expression. The average of all organs and samples at E10.5 was calculated and set to 1. Individual samples were then normalized against this average value (see Materials and Methods for details). Significance was tested between the different organs for each time point. Data are mean ± SEM. * *p* < 0.05, ** *p* < 0.01, *** *p* < 0.001.

Expression of the mRNAs of the three genes *p16*, *p19* and *p21* was assessed at different ages (E10.5, E12.5, E14.5, E16.5, E18.5, P1, P7, P21, 3 months (adult), and 16–18 months (old)). Experiments were conducted on brain, heart, kidney, and liver tissues from which RNA samples were extracted and quantified by reverse transcription-quantitative PCR, normalized to the respective means of *Rplp0*, *Gapdh*, and *β-actin* housekeeping genes. The results below show the comparison of relative expression levels at all investigated ages in each organ for the three genes of interest (Figure 1) and the comparison of the expression levels of each gene in the different organs at each age (Figure 2).

In the brain, we observed a significant upregulation of *p16* expression beginning at E14.5 until P7 compared to E10.5. Surprisingly, *p16* expression dropped significantly at P21 compared to P7 (*p* < 0.05) to reach the highest levels in old animals. *p21* levels increased significantly around E16.5 during embryonic development and remained at stable levels during further development, increasing less than *p16* in brains from old mice. *p19* expression became upregulated around E14.5 and remained more or less stable during further brain development, showing an increase only in brains of old subjects.

Also in the heart, kidney, and liver *p16* expression increased constantly over time with higher expression levels than *p19* and *p21*, which both showed rather low, fluctuating expression during embryonic and postnatal development. Interestingly, in the heart, *p16* tended to drop between P7 and P21 (*p* = 0.070) comparable to the time course in the developing brain. In old stages, *p16* expression in brain, heart, kidney, and liver was largely increased. Also, *p19* and *p21* levels were upregulated in the respective organs, but to a much lesser extent than p16 (Figure 1).

To further analyze the relative mRNA expression data for *p16*, *p19*, and *p21* in embryonic development and in postnatal stages, we compared the expression of each gene in the brain, heart, kidneys, and liver at each time point (Figure 2). Expression levels of *p16* were significantly higher at E10.5 in the brain compared to the developing heart, kidney, and liver. At E14.5, E18.5, and P21, the liver displayed the highest *p16* expression compared to the other investigated organs. At adult and old life stages, p16 expression was high, but not significantly different in the four organ systems studied. *P19* expression did not vary much between brain, heart, kidneys, and liver. An increase of *p19* could be observed in the kidney during development at embryonic day E12.5. Therefore, although p16 and *p19* are situated in the same chromosomal region, spatiotemporal expression patterns seem to be unrelated. Expression of *p21* increased mostly in the brain during embryonic and postnatal development beginning at E16.5 compared to E10.5, while in the other organs, only temporary very restricted significant alterations were observed. Only in old animals, in all organs a significant increase in *p21* expression was noted (Figure 1). At embryonic day 12.5, *p21* expression was highest in the kidneys, while at E16.5 and P21, it was highest in the brain, compared to heart, kidneys, and liver. Even in old animals, *p21* mRNA levels were elevated in brains compared to the kidneys (Figure 2).

### 3.2. Immunohistochemical Investigation of p16 Expression

**Figure 3:**
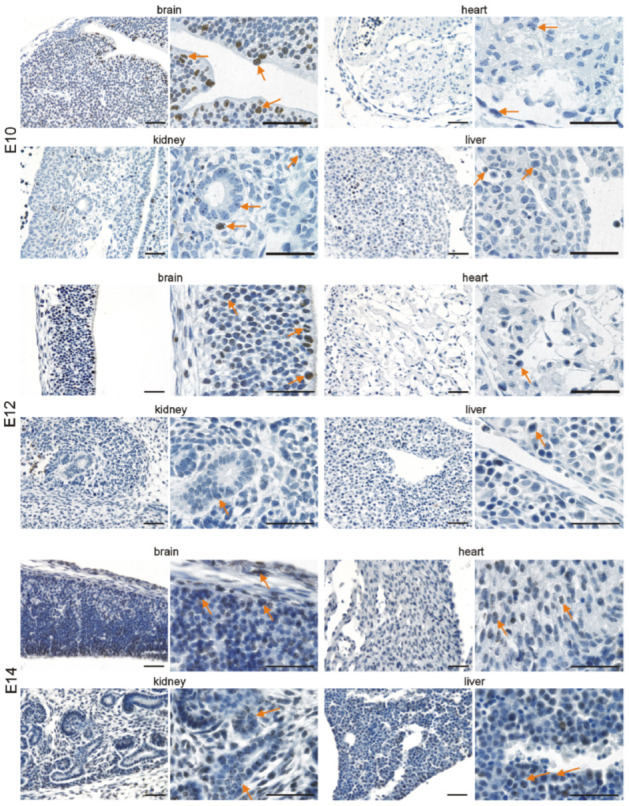
p16 is expressed in the brain, heart, kidneys, and liver during embryonic development (E10–E14). Representative photomicrographs of p16 immunostaining on sections of mouse embryos (3,3′ diaminobenzidine (DAB) substrate, brown, hematoxylin counterstaining) at different stages before birth. Arrows indicate exemplary p16-positive cells. Scale bars represent 50 µm.

**Figure 4:**
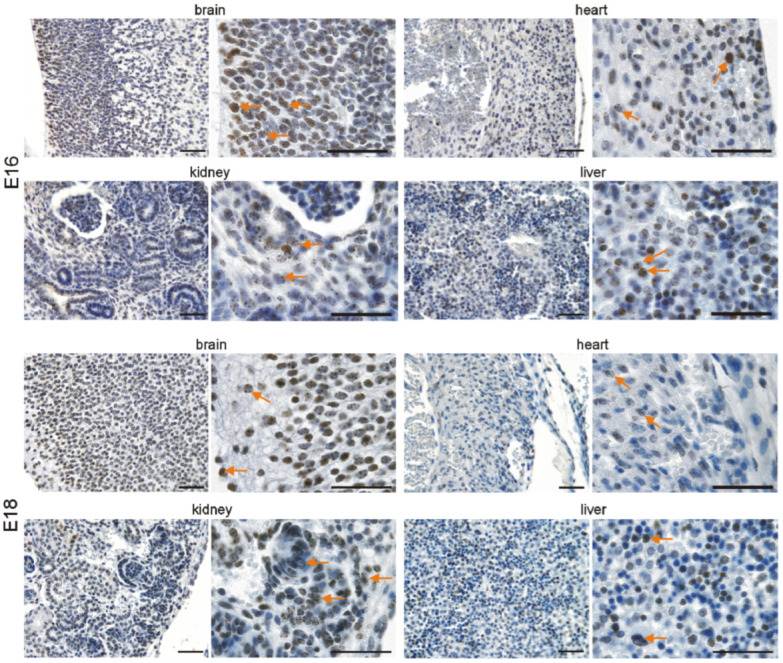
p16 is expressed in the brain, heart, kidneys, and liver during embryonic development (E16–E18). Representative photomicrographs of p16 immunostaining on sections of mouse embryos (3,3′ diaminobenzidine (DAB) substrate, brown, hematoxylin counterstaining) at different stages before birth. Arrows indicate exemplary p16-positive cells. Scale bars represent 50 µm.

**Figure 5:**
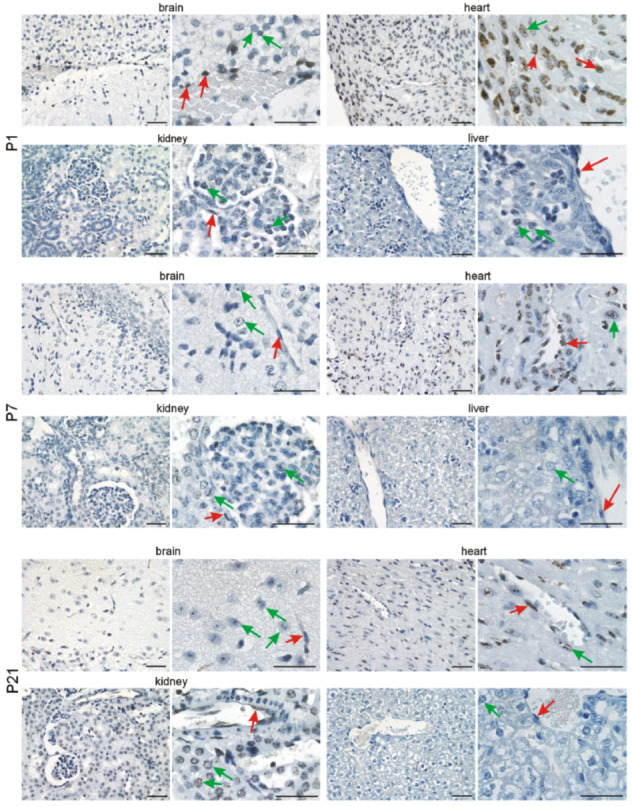
p16 is continuously expressed after birth in vascular and organ cells. Representative photomicrographs of p16 immunostaining for the brain, heart, kidneys, and liver (3,3′ diaminobenzidine (DAB) substrate, brown, hematoxylin counterstaining) at different stages after birth. Note the persistent expression of p16 in neuronal cells of the brain, cardiomyocytes, tubular and glomerular kidney cells, and hepatocytes (green arrows) and endothelial cells (red arrows). Scale bars indicate 50 µm.

**Figure 6:**
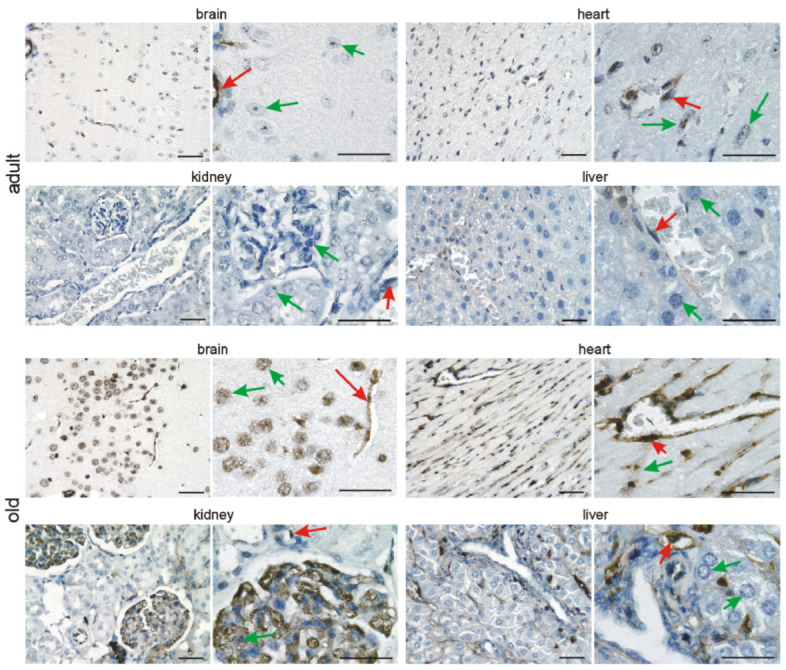
p16 is continuously expressed in adults and increases in old animals. Representative photomicrographs of p16 immunostaining for the brain, heart, kidneys, and liver (3,3′ diaminobenzidine (DAB) substrate, brown, hematoxylin counterstaining). Note the persistent expression of p16 in neuronal cells of the brain, cardiomyocytes, tubular and glomerular kidney cells, and hepatocytes (green arrows) and endothelial cells (red arrows), which increases with age. Scale bars indicate 50 µm.

In addition to quantitative *p16* assessment on the mRNA level, we investigated its expression in the brain, heart, kidneys, and liver at the different time points by immunohistochemistry. In the developing brain, we detected p16 in neuronal cells of the cephalic mesenchyme (E10.5) and the neopallial cortex (E12.5–E18.5). The number of p16-positive neurons increased with differentiation of the brain up to E18.5. (Figure 3 and Figure 4). Neurons of the cortex of old animals displayed a high p16 reactivity. Endothelial cells of the cortex occasionally showed a faint p16 signal (Figure 5), which increased at adult (3 months) and old (16–18 months) stages (Figure 6). Some cardiomyocytes showed p16 expression at early embryonic stages E10.5–E12.5 (Figure 3). With compaction of the myocardium, the number of p16 expressing cardiomyocytes increased from E14.5 to P1. From P7 on, the frequency of p16 expressing cardiomyocytes decreased (Figure 3, Figure 4 and Figure 5). Endothelial cardiac cells frequently showed p16 expression, with a strong increase in old animals (Figure 6). Similarly to the brain and the heart, more p16-positive cells were found upon differentiation of the kidney. Whereas only faint expression of p16 could be detected in the ureteric bud at E10.5 and E12.5, during formation of the metanephric nephrons and interstitial mesenchyme, a high number of cells expressed p16. The number of p16 expressing cells in the kidney decreased postnatally (Figure 3, Figure 4 and Figure 5). In old mice, p16 was highly expressed in glomerular structures, composed of podocytes, fibroblasts, and endothelial cells, and in vessels of the kidney (Figure 6). In the hepatic primordium (E10.5–E12.5), very few cells exhibited p16 expression. With the onset of hepatic hematopoiesis, the number of p16 expressing cells in the embryonic liver increased (Figure 3). From P1 on, when the bone marrow becomes the dominant hematopoietic organ, very few cells in the liver expressed p16 (Figure 5). In livers of old mice, we detected a strong signal in endothelial cells compared to hepatocytes (Figure 6). Liver sections with omission of the primary antibody and sections from p16 knockout mice were used as negative controls for the immunostaining (Figure S1). Embryonic p16 expression was confirmed using a different monoclonal antibody (Figure S2).

### 3.3. Selected SASP Factor Expression

**Figure 7:**
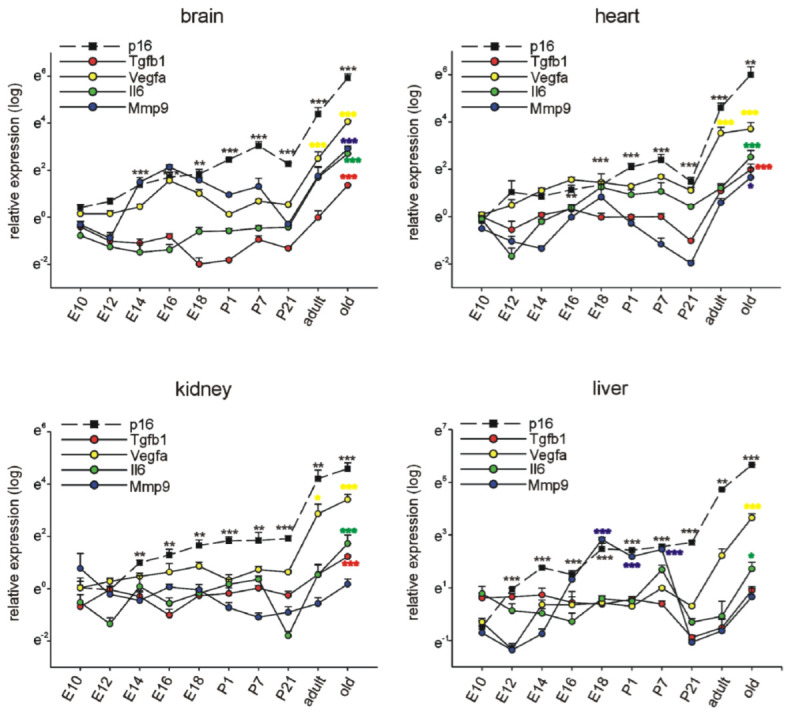
Differential spatiotemporal expression of SASP factors in comparison to *p16*. Quantitative RT-PCRs for *p16*, *Tgfb*, *Vegfa*, *Il6*, and *Mmp9* in mouse brains, hearts, kidneys, and livers at different time points of development and in adulthood (*n* = 4 each, the four samples for E10.5 were each pooled from 7 organs, at E12.5 and 14.5 the four samples were pooled from four organs each). Expression of each gene was normalized to the respective *Gapdh, actin*, and *Rplp0* expression. Next, the average of all organs and samples at E10.5 was calculated. Individual samples were then normalized against this average value. Significance was tested for all time points between E10.5 and 18 months. E: embryonic day, P: postnatal day, adult: 3 months of age, old: 16–18 months of age. Data are mean ± SEM. * *p* < 0.05, ** *p* < 0.01, *** *p* < 0.001.

To gain additional insights into the potential relevance and function of *p16* expression during embryonic and postnatal development and in adult and old mice, we measured mRNA expression of *Il-6*, *Mmp9*, *Tgfb1*, and *Vegfa* as selected SASP (senescence associated secretory phenotype) factors [21,62,63] during the time points and in the organs mentioned before. As each of these genes has individual functions at different time points and in different organs, we considered only a concomitant modification of the four genes as indicative of SASP. In agreement with the literature, we observed an increase in the measured SASP factors in all organs in old mice (Figure 7). *Mmp9* was transiently upregulated during late embryonic and early postnatal development in the liver, but as the other investigated genes did not follow the same time course, it might not be indicative for SASP, and the increase in *p16* expression during embryonic and postnatal development alone is not indicative for senescence.

### 3.4. Higher p16 Expression in Endothelial versus Non-Endothelial Cells in the Liver

**Figure 8:**
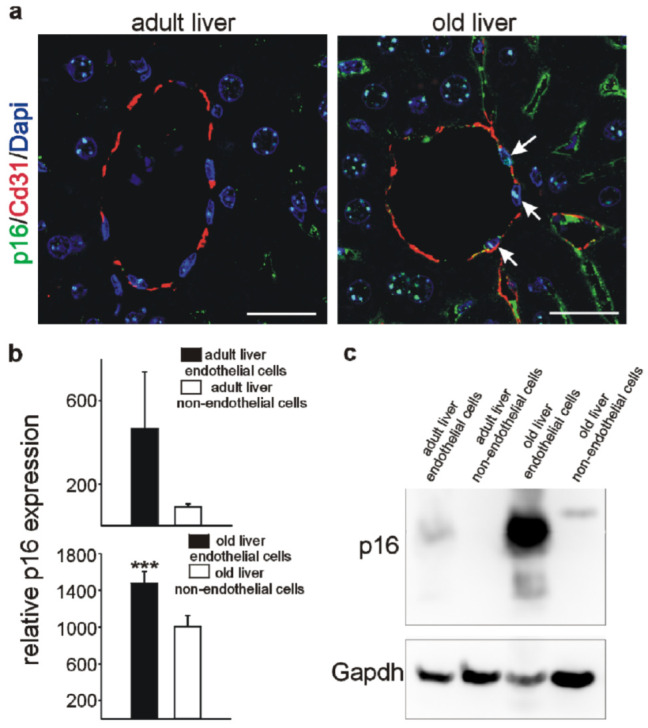
Liver vascular cells express higher levels of p16 than liver cells with aging. (**a**) Confocal images of Cd31 (red)/p16 (green) double-labeling on adult (3 months) (left image) and old (18 months) (right image) liver tissues. Arrows indicate p16/Cd31-positive vascular cells. Scale bars represent 50 µm. (**b**) Quantitative RT-PCRs for *p16* of sorted liver endothelial cells (black bar) and liver organ cells (white bar) at 3 months (upper panel) and 18 months (lower panel). Expression of p16 was normalized to the respective *Gapdh, actin*, and *Rplp0* expression. Data are mean ± SEM. *** *p* < 0.001. (**c**) Western Blot for p16 in 3- or 18-month-old liver endothelial cells and 3- or 18-month-old liver cells. Gapdh served as standard.

As our immunohistochemistry approach suggested higher p16 expression in endothelial versus non-endothelial cells in the liver, we confirmed colocalization of p16 with Cd31 by double-labelling and confocal imaging of wild-type mice livers at 3 and 18 months of age (Figure 8a). For quantitative determinations, endothelial and organ liver cells were isolated from adult (3 months) and old (18 months) mice. In adult livers, endothelial vs. non-endothelial p16 mRNA levels tended to be higher, which became highly significant in old livers (Figure 8b). A comparable result was obtained in Western Blot analyses, showing slightly higher p16 expression in endothelial vs. non-endothelial cells in adult livers, and a dramatic increase of p16 expression in endothelial cells from livers of old animals (Figure 8c). Expression levels for p16 mRNA in endothelial versus non-endothelial cells did not differ for the other adult and old organs, except for the hearts of old mice, where organ cells showed higher p16 expression than endothelial cells. Interestingly, although we observed high p16 expression in old liver endothelial cells versus non-endothelial cells, this was not correlated with an increase in the expression of SASP genes except for *Tgfb1*, while *Vegfa* expression was even lower in endothelial cells (Figure S3). This is consistent with the previous observation that liver endothelial cells in aged mice are highly metabolic active and functional despite high p16 expression [64].

## 4. Discussion

Our results have shown dynamic and differential expression of *p16* during embryonic and postnatal development as well as in adult and old mice in the brain, heart, kidneys, and liver. Expression of *p16* was varying significantly within each organ during embryonic development in a matter of days. At the same time, *p19* and *p21* did not show such a remarkable variation of expression. We limited the current study to the investigation of brain, heart, kidney, and liver as these organs already develop at the embryonic time points chosen [65,66,67,68] and are relatively easy to isolate. Nevertheless, it is possible that *p16*, *p19*, and *p21* might be expressed in a variety of developing organs. For example, *p21* expression has already been described during embryonic development, i.e., in muscle, nasal epithelium, tongue muscles, hair follicles, epidermis, and cartilage, and was related in part to growth arrest and senescence [42,69,70]. Expression of *p19* has been described in the developing nervous system [44], while p16 has not been detected during embryonic development in earlier studies [45]. However, the authors of this study did not exclude that p16INK4a mRNA might be expressed at low levels or restricted sites in embryos. The authors reported an upregulation of p16 transcripts in organs from 15 month-old mice; however, the original PCR data do not show a specific p16 signal [45]. Using highly sensitive quantitative RT-PCR and antibody staining methods [48,54,55,56,57,58,71], our finding of a relatively high *p16* expression during development and especially at old stages might be more accurate. 

Upregulation of *p16*/*p19* and *p21* is widely accepted as a marker of aging and senescence [16,72,73,74]. In human tissue samples, P16 was detected in endocrine and exocrine pancreas, skin, kidneys, liver, intestine, spleen, brain, and lung. Its expression increased in all investigated organs except for the lung with increasing age [75]. We demonstrate here that murine *p16* expression highly increased in all organs investigated between 3 months and 16 months of age. We observed a less pronounced increase in *p19* and *p21* compared to *p16* in old versus adult mice, which is consistent with previous reports in mice and humans [76]. We could not detect organ-specific differences in *p16* expression at 16 months of age, which contrasts with a recent study from Yousefzadeh et al. [77]. This might be explained by the age difference of the animals used in their study which compares mice aged from 15–19 weeks with 120-week-old subjects. A study comparing P16 protein expression by immunohistochemistry in human tissues from young, middle-aged, and old donors confirmed a significant increase of P16 in the liver, kidneys, and brain in old subjects. However, no P16 expression could be detected at all ages investigated in the heart, which might be due to species differences [75].

Regarding expression of *p16*, *p19*, and *p21* and the role of senescence during embryonic development, the literature is more controversial. Unlike *p21*, *p16* and *p19* were reported to be absent in early studies as discussed above [45,69]. Senescence, however, has been detected based on SA-β-galactosidase staining during embryonic development, which seems to depend on *p21* expression [42,43,78]. Interestingly, absence of p21 was compensated by apoptosis, but still slight developmental abnormalities were detectable [43]. Although these studies focused mainly on p21, *p16* loss has also been shown to result in developmental defects in the eye [79]; inactivation of *p16* and *p19* induced cardiomyocyte proliferation [80]; p16 has been detected in the ventricular and subventricular zones at embryonic and early postnatal stages in the rat brain; SA-β-galactosidase activity and *p16* expression has been detected in regressing mesonephros of quails [81]; *p16* expression in mouse embryos has been detected in motoneurons and the senolytic ABT-263 decreased the number of these cells [82]. Nevertheless, not all highly p16-positive cells are necessarily senescent [83,84]. For example, overexpression of *p16* slowed cell cycle progression in the G0/G1 phase and induced erythroid lineage differentiation [85], which might correspond to the early p16 expression in embryonic mouse livers [86]. Lack of *p16* is linked to increased cardiomyocyte proliferation [80], while lower cardiomyocyte proliferation, differentiation, and specification are required for myocardial compaction [87,88], which coincides with our observed cardiac p16 expression. 

Furthermore, the notion of senescence as an irreversible form of cell cycle arrest, leading to death of the cell [18] has been recently questioned by the observation that cancer cells can escape from the senescence induced cell cycle arrest and gain a highly aggressive growth potential [89]. Highly interesting, it has also been demonstrated that embryonic senescent cells re-enter the cell cycle and contribute later to tissue formation [40]. We observed organ specific variations of *p16* expression, especially by immunohistochemical localization of p16 protein during development. Expression of p16 in development might reflect its function of slowing down cell cycle progression, a process essential for cell type specific differentiation. Knockout mice for *p16/p19* and selectively for *p16* are prone to tumor development [90,91,92], but potential developmental defects have not been investigated as the mice are viable and fertile. Thus, re-evaluation of potential developmental defects in mice with inactivation of *p16* or elimination of p16 expressing cells remains an interesting challenge for further studies.

In postnatal livers, p16 has been intensively studied. p16 has protective effects in non-alcoholic steatohepatitis and liver fibrosis through the regulation of reactive oxygen species (ROS) and oxidative stress [93,94]. Specific removal of liver endothelial cells expressing high levels of p16 resulted in fibrosis and liver deterioration, indicating that these cells are required for the maintenance of liver physiology [64]. However, detailed future studies using conditional cell type-specific knockout approaches will be needed to determine the specific function of p16 in liver endothelial cells.

## 5. Conclusions

Taken together, p16 expression in embryonic stages might reflect an implication in developmental differentiation processes. Further elucidation of the characteristics of p16 expressing cells, using embryos with inactivation or specific elimination of p16 expressing cells will hopefully shed light on the possible functions of p16 in differentiation, in addition to its implication in senescence and aging. Moreover, in aged mice, the significant upregulation of p16 expression in liver endothelial cells points to a selective role in liver endothelial physiology.

## Figures and Tables

**Table 1:** Primers used for quantitative RT-PCR.

Gene of Interest	Oligonucleotide Sequences	References
*p16ink4*	F: AGGGCCGTGTGCATGACGTG R: GCACCGGGCGGGAGAAGGTA	[59]
*p19arf*	F: CGCTCTGGCTTTCGTGAAC R: GTGCGGCCCTCTTCTCAA	[60]
*p21*	F: AATTGGAGTCAGGCGCAGAT R: CATGAGCGCATCGCAATCAC	[61]
*Tgf-b1*	F: AGCTGGTGAAACGGAAGCG R: GCGAGCCTTAGTTTGGACAGG	This study
*Vegfa*	F: CTCACCAAAGCCAGCACATA R: AATGCTTTCTCCGCTCTGAA	[54]
*Il-6*	F: CACTTCACAAGTCGGAGGCT R: TGCCATTGCACAACTCTTTTCT	[54]
*Mmp9*	F: CCATGCACTGGGCTTAGATCA B: GGCCTTGGGTCAGGCTTAGA	[54]
*Gapdh*	F: AGGTCGGTGTGAACGGATTTG R: TGTAGACCATGTAGTTGAGGTCA	[47,48,54,58]
*β-actin*	F: CTTCCTCCCTGGAGAAGAGC R: ATGCCACAGGATTCCATACC	[47,48,54,58]
*Rplp0*	F: CACTGGTCTAGGACCCGAGAAG R: GGTGCCTCTGGAGATTTTCG	[47,48,54,58]

## Supplementary Materials

The following are available online at www.mdpi.com/article/10.3390/cells11030541/s1, Figure S1: Representative photomicrographs of p16 immunostaining on sections of mouse livers, Figure S2: Representative photomicrographs of p16 immunostaining using a different p16 antibody (clone 1E12E10) on sections of mouse embryos, Figure S3: Expression of selected senescence-associated secretory phenotype (SASP) factors and p16 in endothelial and non-endothelial cells.
