# Supplementary material for: Dynamic Spatiotemporal Expression Pattern of the Senescence-Associated Factor p16Ink4a in Development and Aging
Source: Cells. 2022 Feb 4;11(3):541. doi: 10.3390/cells11030541 (PMC8833900; doi:10.3390/cells11030541)
Supplement: Supplementary file 1 [file cells-11-00541-s001.zip › cells-1575294-supplementary.pdf]

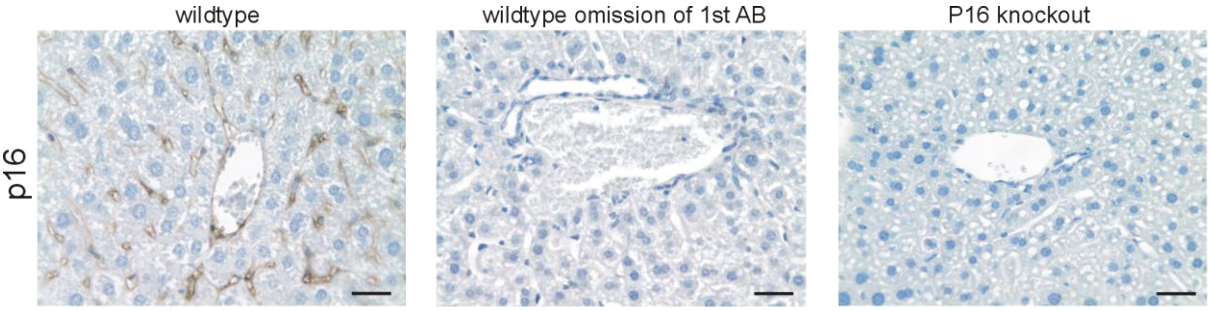

Supplementary Figure S1: Representative photomicrographs of p16 immunostaining on sections of mouse livers (3,3' diaminobenzidine (DAB) substrate, brown, hematoxylin, blue, nuclear counterstaining) showing examples for the staining of a wildtype liver, a section with omission of the first antibody (1st AB), and a p16 knockout liver. Scale bars represent 50µm.

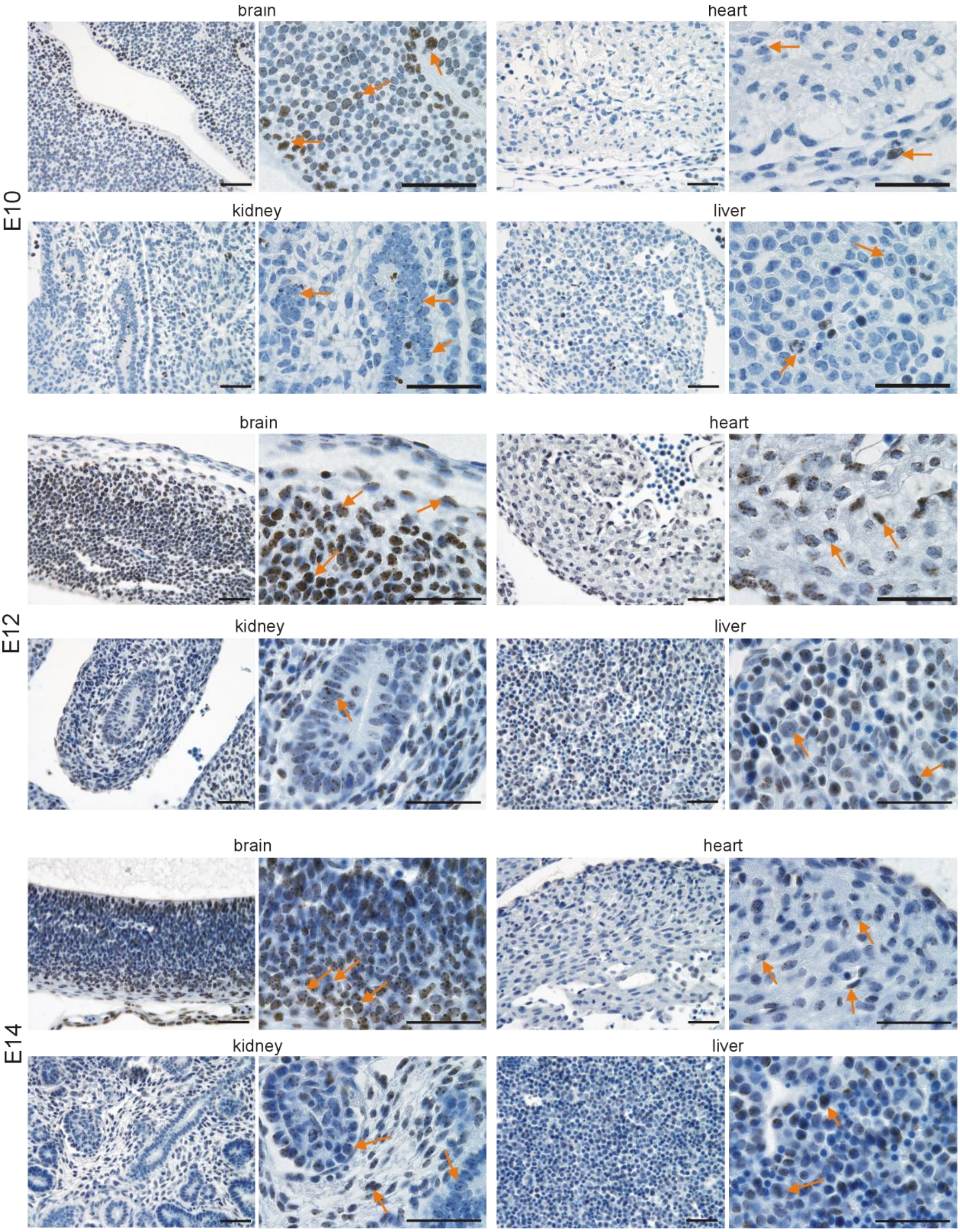
E10
brain
heart
kidney
liver
E12
brain
heart
kidney
liver
E14
brain
heart
kidney
liver

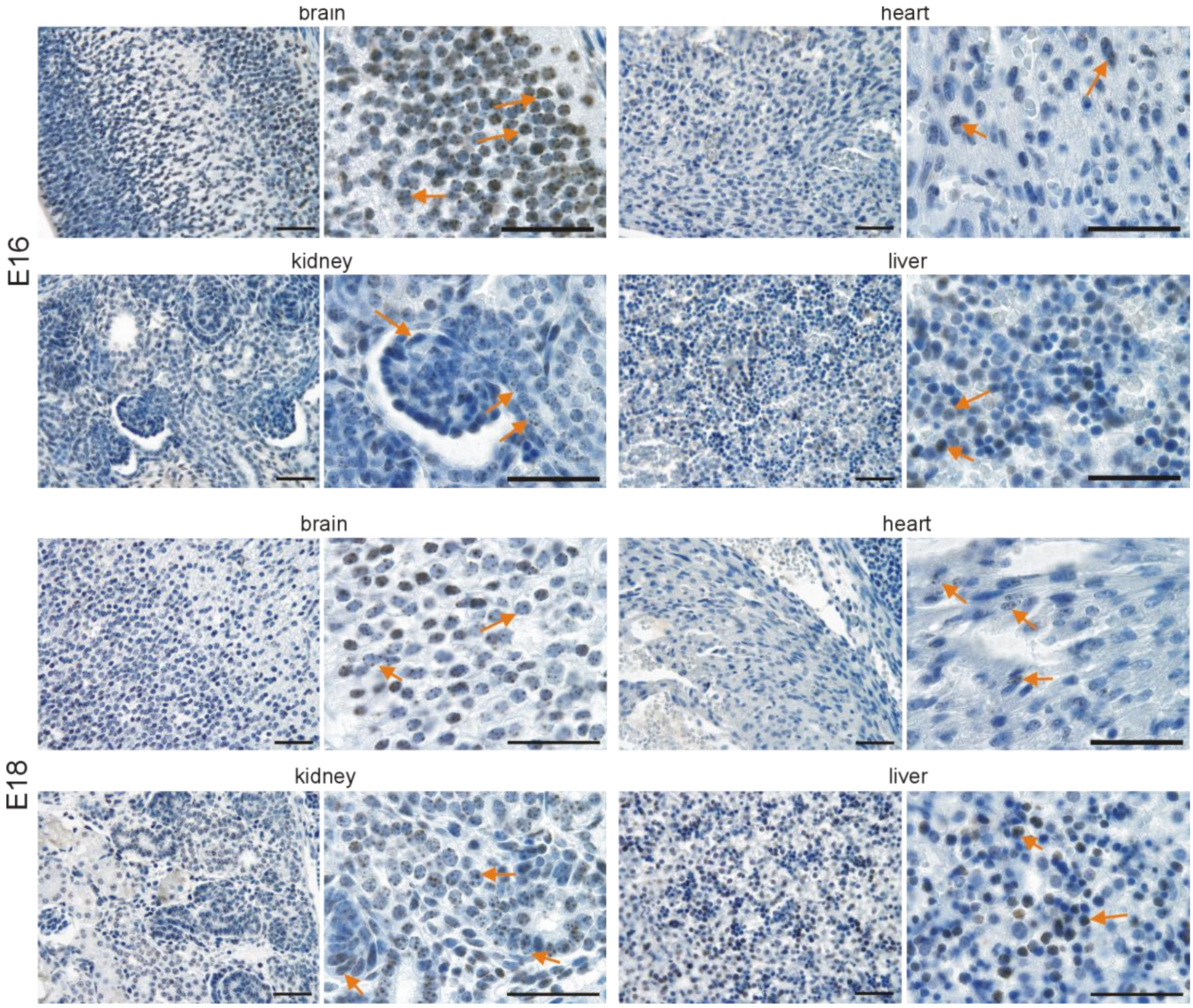

Supplementary Figure S2: Representative photomicrographs of p16 immunostaining using a different p16 antibody (clone 1E12E10) on sections of mouse embryos (3,3' diaminobenzidine (DAB) substrate, brown, hematoxylin counterstaining) at different stages before birth. Arrows indicate examples of p16 positive cells. Scale bars represent 50μm.

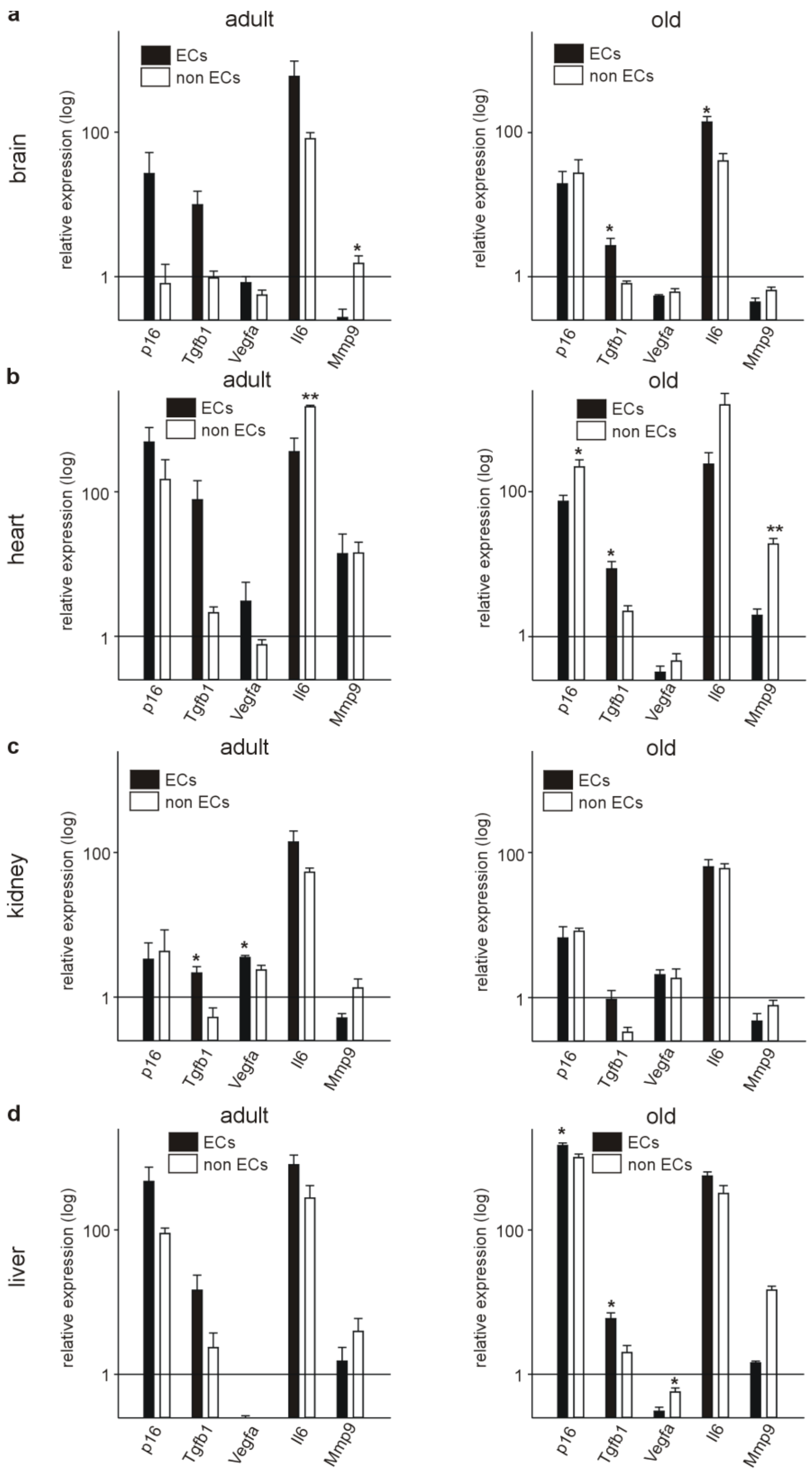

Supplementary Figure S3: Expression of selected senescence-associated secretory phenotype (SASP) factors and p16 in endothelial and non-endothelial cells of different organs from adult (3 months) and old (18 months) mice. Quantitative RT-PCRs for p16, Tgfb, Vegfa, Il6, and Mmp9 in mouse brains, hearts, kidneys, and livers. Expression of each gene was normalized to the respective *Gapdh, actin, and Rplp0* expression. The average of all organs and samples at E10.5 was calculated and set to 1. Individual samples were then normalized against this average value (see Materials and Methods for details). Significance was tested for each gene between endothelial and non-endothelial cells. Data are mean ± SEM. *$p<0.05$, **$p<0.01$.
